# Dr. Samuel Lilienthal

**Published:** 1891-11

**Authors:** 


### DR. SAMUEL LILIENTHAL.

Dr. Samuel Lilienthal, who was the oldest homœopathic physician in America, died Friday evening, October 2d, at the residence of his son, Dr. James E. Lilienthal, 1316 Van Ness Avenue, San Francisco, California, aged seventy-five years ten months and twenty-eight days. The deceased was a well-known authority on medical matters, and enjoyed a national reputation as a writer. The immediate cause of death was heart disease, from which deceased had been a sufferer for some time past.

Dr. Lilienthal was born in Munich, Bavaria, on November 5th, 1815, and graduated at the University of Munich in 1838. He emigrated to New York, with his distinguished brother, Rev. Dr. Lilienthal, of Cincinnati, and located in the Empire State, where he soon was recognized as one of the leading physicians.

Deceased was appointed professor of mental and nervous diseases in the New York Homœopathic College, and professor of clinical medicine in the New York College for Women.

He was a great advocate and determined friend of women, and is responsible for the success of many of the excellent female physicians of this country.

As a writer Dr. Lilienthal was very prolific on all subjects pertaining to his favorite science, and was the author of *Homœpathic Therapeutics*, of which three editions have been published, and he was at work on the fourth at the time of his death.

Though he would sometimes jestingly call himself "a mongrel" in his private letters to the editor of this journal, yet his book of therapeutics does not show any such medical views. On the contrary, it is filled with well-selected characteristics of each remedy under the name of the disease, after the manner of Dr. Guernsey's *Obstetrics* and Rane's *Special Pathology and Therapeutic Hints*.

Dr. Lilienthal's translations are well known to the readers of THE HOMŒOPATHIC PHYSICIAN. He was the fast friend of this journal, and supplied it with a large number of contributions, many of which we have still on file, not having had space enough to publish them as fast as furnished.

He was the oldest living practitioner of Homœopathy in the United States, was for many years editor of the *North American Journal of Homœopathy*, and was the recipient from the University of Munich, in the year 1888, of a fifty-year diploma, which is considered a very great and honorable distinction, and given only in rarest instances for most honorable practice. He was a friend of the poor and needy, and many thousands of the poor in the great city of New York will feel that one of their best friends has passed away.

He came to San Francisco some six years since, having retired from practice several years before that time, to be with his family, who are all residents of that city. He made his home with his son, Dr. James E. Lilienthal, on Van Ness Avenue, an active practitioner. The other members of the family are Mr. E. R. Lilienthal and J. L. Lilienthal, of the well-known firm of Lilienthal & Co., commission merchants, on Front Street. During his residence in San Francisco he did not practice at all, having entirely retired from public life and spending all of his time with his sons' families.

